# Invasive and Native Plants Differentially Respond to Exogenous Phosphorus Addition in Root Growth and Nutrition Regulated by Arbuscular Mycorrhizal Fungi

**DOI:** 10.3390/plants12112195

**Published:** 2023-06-01

**Authors:** Xionggui Yang, Kaiping Shen, Tingting Xia, Yuejun He, Yun Guo, Bangli Wu, Xu Han, Jiawei Yan, Min Jiao

**Affiliations:** 1 Forestry College, Research Center of Forest Ecology, Guizhou University, Guiyang 550025, China; yxg99512@163.com (X.Y.); skp0825@163.com (K.S.); xtt1268@163.com (T.X.); zihanyun2013@163.com (Y.G.); wubangli1116@163.com (B.W.); hanxukumo@163.com (X.H.); jiaweiyan1017@163.com (J.Y.); jiaomin999@163.com (M.J.); 2 College of Eco-Environmental Engineering, Guizhou Minzu University, Guiyang 550025, China

**Keywords:** plant invasion, arbuscular mycorrhizal fungi, phosphorus addition, competition, root trait

## Abstract

Plant invasion has severely damaged ecosystem stability and species diversity worldwide. The cooperation between arbuscular mycorrhizal fungi (AMF) and plant roots is often affected by changes in the external environment. Exogenous phosphorus (P) addition can alter the root absorption of soil resources, thus regulating the root growth and development of exotic and native plants. However, it remains unclear how exogenous P addition regulates the root growth and development of exotic and native plants mediated by AMF, affecting the exotic plant invasion. In this experiment, the invasive plant *Eupatorium adenophorum* and native plant *Eupatorium lindleyanum* were selected and cultured under intraspecific (Intra-) competition and interspecific (Inter-) competition conditions, involving inoculation with (M^+^) and without AMF (M^−^) and three different levels of P addition including no addition (P_0_), addition with 15 mg P kg^−1^ soil (P_15_), and addition with 25 mg P kg^−1^ soil (P_25_) for the two species. Root traits of the two species were analyzed to study the response of the two species’ roots to AMF inoculation and P addition. The results showed that AMF significantly promoted the root biomass, length, surface area, volume, tips, branching points, and carbon (C), nitrogen (N), and P accumulation of the two species. Under M^+^ treatment, the Inter- competition decreased the root growth and nutrient accumulation of invasive *E. adenophorum* but increased the root growth and nutrient accumulation of native *E. lindleyanum* relative to the Intra- competition. Meanwhile, the exotic and native plants responded differently to P addition, exhibiting root growth and nutrient accumulation of invasive *E. adenophorum* increased with P addition, whereas native *E. lindleyanum* reduced with P addition. Further, the root growth and nutrition accumulation of native *E. lindleyanum* were higher than invasive *E. adenophorum* under Inter- competition. In conclusion, exogenous P addition promoted the invasive plant but reduced the native plant in root growth and nutrient accumulation regulated by AMF, although the native plant outcompeted the invasive plant when the two species competed. The findings provide a critical perspective that the anthropogenic P fertilizer addition might potentially contribute to the successful invasion of exotic plants.

## 1. Introduction

Exotic plant invasions can seriously threaten local communities’ species diversity and stability, causing substantial economic losses and ecological damage [1,2]. An essential factor in successfully invading alien plants is their functional traits, such as root traits [3]. The underground competition of plants mainly depends on the root system, and the root morphological character can reveal the competition advantage differences between alien and native plants [4]. Research showed that higher root trait values could improve the competitive advantage of exotic plants, thus obtaining more nutrients for growth than native species under the same conditions, which benefits them in adapting to new habitats and spreading faster [5,6]. For example, Broadbent, Stevens [7] showed that the ability of invasive grasses to outcompete native grasses for below-ground resources may be related to their greater root biomass relative to native grasses. Nevertheless, root biomass is unlikely to determine the outcome of below-ground competition between species and usually interacts with other factors, such as feedback with soil biota [8]. In addition, studies reveal that having more root hairs or root branches allows roots to explore larger soil volumes and that increased surface area of root hairs or branches facilitates the release of root-derived organics and phosphate, thus, increasing nutrient acquisition efficiency [9,10]. Betekhtina, Ronzhina [11] suggested that alien *Heracleum sosnowskyi* had more root branches and root hair development than congeneric native *H. sibiricum*, leading to a higher nutrient absorption efficiency of the invader than native, thus helping the invasive *H. sosnowskyi* win the competition with the native *H.sibiricum*. Therefore, root traits play a vital role in the competition between alien and native plants. In invasion ecology, nevertheless, the majority of trait-based comparisons are concentrated on the more accessible above-ground plant traits [4,12]. Thus, more research is necessary about how below-ground root traits determine the competitive advantage of exotic and native plants.

Below-ground root competition between exotic and native plants is primarily impacted by soil nutrient availability changes [7]. Generally, soil phosphorus (P) fluctuations caused by human activities can drive variations in soil nutrient availability. P is a necessary constant nutrient for plant growth, and thus, P deficiency can affect plant growth to a large extent [13]. Thus, it can be reasonably assumed that changes in soil P elements affect the invasion of alien plants [14]. Recent research discovered that the richness of exotic plants increased with increasing P concentration [15]. Additionally, Chen, Zhang [16] suggested that P addition significantly increased the root length, root biomass, and nutrient accumulation of exotic *Flaveria bidentis*, resulting in a higher competitive advantage. However, P is difficult for plants to obtain as most soil P is bound to the surface of molecules or minerals and has low mobility, thus limiting plants’ growth [17]. Given the difficulty of moving P in the soil, plants, including invasive and native plants, invariably change their root morphological traits and activate insoluble phosphorus in the soil by secreting activating enzymes, organic acids, protons, and other substances from the root system to actively approach and compete for more P elements [8,18,19,20]. In addition to relying on their root systems, many exotic and native plants improve their accessibility to P elements by establishing partnerships with various microorganisms in the soil.

Arbuscular mycorrhizal fungi (AMF) are a class of soil microorganisms that have long been in a mutually beneficial symbiotic partnership with plants and have been extensively studied, including invasion ecology [21,22]. AM fungi are ubiquitous and can form symbiotic associations with over 80% of terrestrial plants, which facilitate the host plants’ growth by helping plant roots absorb mineral nutrients such as P from the soil; as a reward, the host plant supplies a carbon (C) source from the AM fungi to sustain hyphal development [21]. For instance, AM fungi can induce host plants to release root exudates, such as organic acids and phosphatases, to mineralize organic P in the soil and increase the activity of phosphatases, creating a more favorable growth environment for plants [23]. Plants mainly absorb soil-available phosphatase (AP) through their root systems; plant–mycorrhizae symbiosis can help plants to absorb soil AP [24]. Studies have shown that P uptake directly through roots requires more plant resource investment than P acquisition driven by the AMF–plant symbiosis [25]. Thus, the P accessibility of exotic and native plants by combining with AMF may directly contribute to the competitive differences between the two species. For example, Cheng, Yue [26] showed that AMF improved the competitiveness of native *Bidens biternata* by promoting P absorption, helping natives to resist the alien *Bidens alba*. Additionally, Sun, Yang [27] concluded that AMF probably received more myristic acids in return from exotic Asteraceae plants than native Asteraceae plants, contributing to the P absorption capacity of invaders over natives. Further, in addition to directly utilizing hyphae, AMF can aid plants in nutrient acquisition by altering root morphology. For instance, AMF can change the root morphological characteristics of invasive *Microstegium vimineum* and increase P absorption capacity, thus helping invaders to invade successfully [28]. Additionally, the epitaxial root hyphae formed by AMF can improve root length, surface area, and volume, which promotes plants to obtain more nutrients for growth in nutrient-deficient karst areas [29]. However, how AMF associated with exogenous P addition affects the competition direction between alien and native plants through regulating root morphological developments and nutrition acquisition remains unclear.

Indeed, with increased human activities, such as excessively added P fertilizer, the soil P availability is inordinately altered [30]. The plant root is the most sensitive organ to variations in soil nutrient availability [31]. However, to date, empirical evidence of the impacts of changes in P resource availability on the competitive interactions of root systems between exotic and native plants is scarce. AMF–plant symbiosis often enhances the nutrient competitiveness of alien plants, resulting in greater growth of invaders than natives [32,33]. In addition, the root morphological characteristics can characterize the plant’s nutrient acquisition capacity [34,35]. Therefore, we hypothesized that (1) AMF promotes the root growth and development of invasive plants more than native plants, resulting in a higher nutrient acquisition ability for invasive plants than native plants (H1). Moreover, many studies suggest that as soil nutrient content increases, such as the P element, plants reduce their dependence on AMF, thus affecting root morphological traits [36,37]. Therefore, we hypothesized that (2) with exogenous P increases, root mycorrhizal colonization of invasive and native plants is reduced, and the two species’ root growth and nutrition acquisition increase (H2). According to Xia, Wang [3], AMF can confer higher competitiveness to invaders by promoting superior root growth and nutrient absorption of invasive plants over native plants. Meanwhile, P addition improved the root growth of invasive plants, and appropriate P addition is beneficial for AMF to promote plant root growth of invasive plants over native plants [16,38]. Consequently, we hypothesized that (3) exogenous P addition promotes higher root growth and nutrient acquisition of invasive plants than native plants regulated by AMF (H3). To test the three hypotheses, we conducted a competitive experiment using an alien species, *Eupatorium adenophorum,* and a native species, *Eupatorium lindleyanum*. The aim was to explore how external P addition altered the root morphological characteristics of the two plants regulated by AMF, thus affecting the invasion of alien species.

## 2. Results

### 2.1. The Root Mycorrhizal Colonization of Alien Plant E. adenophorum and Native Plant E. lindleyanum

**Figure 1:**
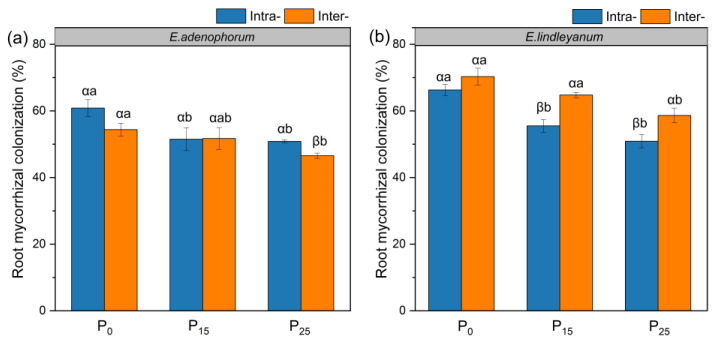
The root mycorrhizal colonization of alien plant *E. adenophorum* and indigenous plant *E. lindleyanum*. The subgraph (**a**,**b**) indicate the root mycorrhizal colonization of alien plant *E. adenophorum* and indigenous plant *E. lindleyanum*, respectively. Abbreviations: Intra- = intraspecific, Inter- = interspecific; P_0_ = without P addition of 0 mg·kg^−1^, P_15_ = with P addition of 15 mg·kg^−1^, P_25_ = with P addition of 25 mg·kg^−1^. Different Greek letters (α, β) above the bars indicate significant differences between Intra- and Inter- treatments; different lowercase letters (a, b) above the bars indicate significant differences between P_0_, P_15,_ and P_25_ treatments (*p* < 0.05). Note: As the uninoculated plant roots were observed to be colonized by AMF, meaning that the root mycorrhizal colonization under the M^−^ treatment was considered to be zero, we only show the root mycorrhizal colonization under AMF inoculation in the figure.

The exotic plant *E. adenophorum* and the native *E. lindleyanum* showed high root mycorrhizal colonization after (40–76.2%) inoculation with AMF (Figure 1a,b). Under P_15_ and P_25_ conditions, the root mycorrhizal colonization in Inter- competition was significantly higher than in Intra- competition for native *E. lindleyanum* (Figure 1b). In addition, the root mycorrhizal colonization under the P_0_ condition was higher than the P_15_ and P_25_ conditions in Intra- and Inter- competition for the two species (Figure 1a,b). Overall, the root mycorrhizal colonization of both species gradually decreased with increasing P addition under Intra- and Inter- competition.

### 2.2. The Root Biomass of Alien Plant E. adenophorum and Native Plant E. lindleyanum

**Figure 2:**
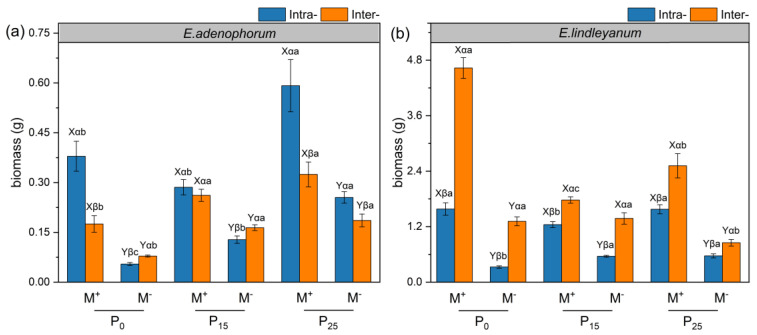
The root biomass of alien plant *E. adenophorum* and indigenous plant *E. lindleyanum*. The subgraph (**a**,**b**) indicate the root biomass of alien plant *E. adenophorum* and indigenous plant *E. lindleyanum*, respectively. Abbreviations: M^+^ = with AMF; M^−^ = without AMF; Intra- = intraspecific, Inter- = interspecific; P_0_ = without P addition of 0 mg·kg^−1^, P_15_ = with P addition of 15 mg·kg^−1^, P_25_ = with P addition of 25 mg·kg^−1^. Different capital letters (X, Y) above the bars indicate significant differences between M^+^ and M^−^ treatments; different Greek letters (α, β) above the bars indicate significant differences between Intra- and Inter- treatments; different lowercase letters (a–c) above the bars indicate significant differences between P_0_, P_15_, and P_25_ treatments (*p* < 0.05).

The AMF, competition, P addition treatments, and the interaction of C × P and M × C × P had a remarkable impact on the root biomass of the alien plant *E. adenophorum* and native plant *E. lindleyanum* (Table S1). Under three P addition conditions, AMF promoted the root biomass of the two species under Intra- and Inter- competition (Figure 2a,b). For alien *E. adenophorum* with M^−^ treatment, contrary to the P_25_ condition, the root biomass in Inter- was significantly higher than in Intra- under P_0_ and P_15_ conditions; with M^+^ treatment, the root biomass in Intra- was remarkably higher than in Inter- under P_0_ and P_25_ conditions (Figure 2a). For native *E. lindleyanum* with M^−^ and M^+^ treatments, the root biomass in Inter- was remarkably higher than in Intra- under three P addition conditions (Figure 2b). For alien *E. adenophorum* with M^−^ and M^+^ treatments, the root biomass under the P_25_ condition was higher than under the P_0_ and P_15_ conditions in Intra- and Inter- competition (Figure 2a). For native *E. lindleyanum* with M^+^ treatment, the root biomass under the P_15_ condition was remarkably lower than under the P_0_ and P_25_ conditions in Intra- and Inter- competition (Figure 2b). Overall, regardless of Intra- and Inter- competition, AMF significantly benefited the root biomass of alien *E. adenophorum* and native *E. lindleyanum*, and exogenous P addition had different effects on the two species.

### 2.3. The Root Traits of Alien Plant E. adenophorum and Native Plant E. lindleyanum

**Figure 3:**
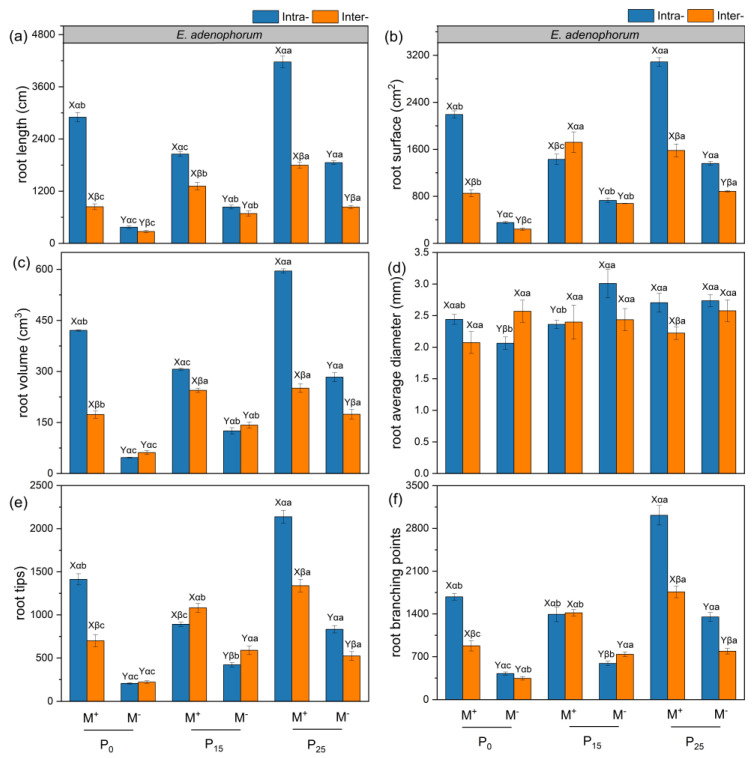
The root traits of alien plant *E. adenophorum*. The subgraph (**a**–**f**) indicate the root length, root surface, root volume, root average diameter, root tips, root branching points of alien plant *E. adenophorum*, respectively. Abbreviations: M^+^ = with AMF; M^−^ = without AMF; Intra- = intraspecific, Inter- = interspecific; P_0_ = without P addition of 0 mg·kg^−1^, P_15_ = with P addition of 15 mg·kg^−1^, P_25_ = with P addition of 25 mg·kg^−1^. Different capital letters (X, Y) above the bars indicate significant differences between M^+^ and M^−^ treatments; different Greek letters (α, β) above the bars indicate significant differences between Intra- and Inter- treatments; different lowercase letters (a–c) above the bars indicate significant differences between P_0_, P_15_, and P_25_ treatments (*p* < 0.05).

**Figure 4:**
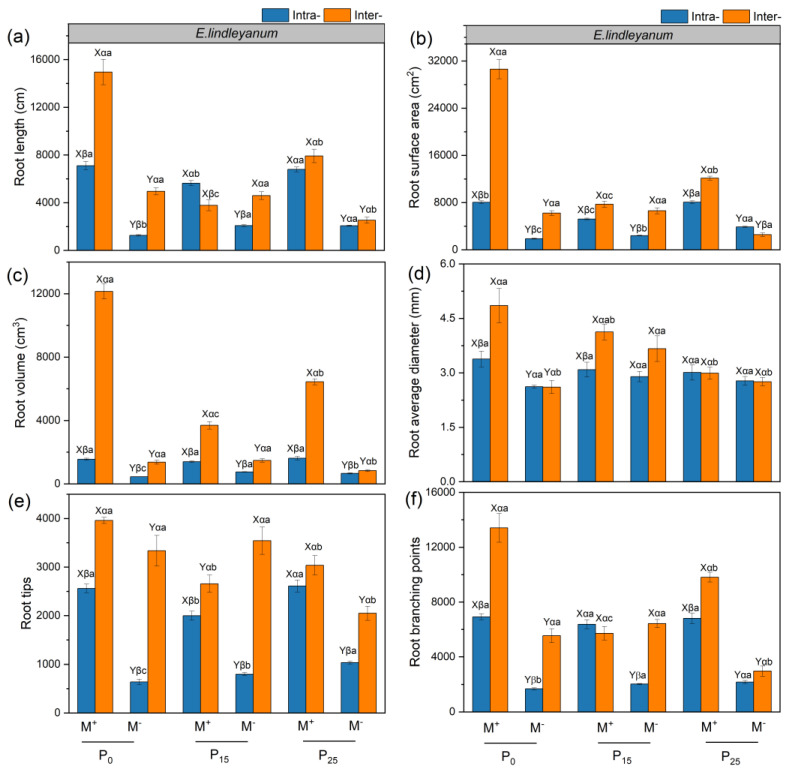
The root traits of the indigenous plant *E. lindleyanum***.** The subgraph (**a**–**f**) indicate the root length, root surface, root volume, root average diameter, root tips, root branching points of indigenous plant *E. lindleyanum*, respectively. Abbreviations: M^+^ = with AMF; M^−^ = without AMF; Intra- = intraspecific, Inter- = interspecific; P_0_ = without P addition of 0 mg·kg^−1^, P_15_ = with P addition of 15 mg·kg^−1^, P_25_ = with P addition of 25 mg·kg^−1^. Different capital letters (X, Y) above the bars indicate significant differences between M^+^ and M^−^ treatments; different Greek letters (α, β) above the bars indicate significant differences between Intra- and Inter- treatments; different lowercase letters (a–c) above the bars indicate significant differences between P_0_, P_15_, and P_25_ treatments (*p* < 0.05).

The AMF, competition, P addition treatments, and their interaction generally influenced the root length, surface area, volume, average diameter, tips, and branching points of the alien plant *E. adenophorum* and native plant *E. lindleyanum* (Table S1). Under Inter- competition, AMF had a remarkable impact on root length, surface area, volume, tips, and branching points of the two species under P_0_ and P_25_ addition conditions, and AMF significantly enhanced the average root diameter of native *E. lindleyanum* under P_0_ condition (Figure 3a–f and Figure 4a–f). Contrary to native *E. lindleyanum*, the root length, surface area, volume, average diameter, tips, and branching points of alien *E. adenophorum* with M^+^ treatment in Intra- was higher than in Inter- under P_0_ and P_25_ conditions (Figure 3a–f and Figure 4a–f). For alien *E. adenophorum* with M^−^ and M^+^ treatments, the root traits under P_25_ conditions were higher than P_0_ and P_15_ conditions in Intra- and Inter- competition except for the root average diameter (Figure 3a–f). For native *E. lindleyanum* with M^+^ treatment, the root traits under P_0_ treatment were remarkably higher than those of P_15_ and P_25_ conditions in Inter- competition (Figure 4a–f).

### 2.4. The Specific Root Traits of Alien Plant E. adenophorum and Native Plant E. lindleyanum

**Figure 5:**
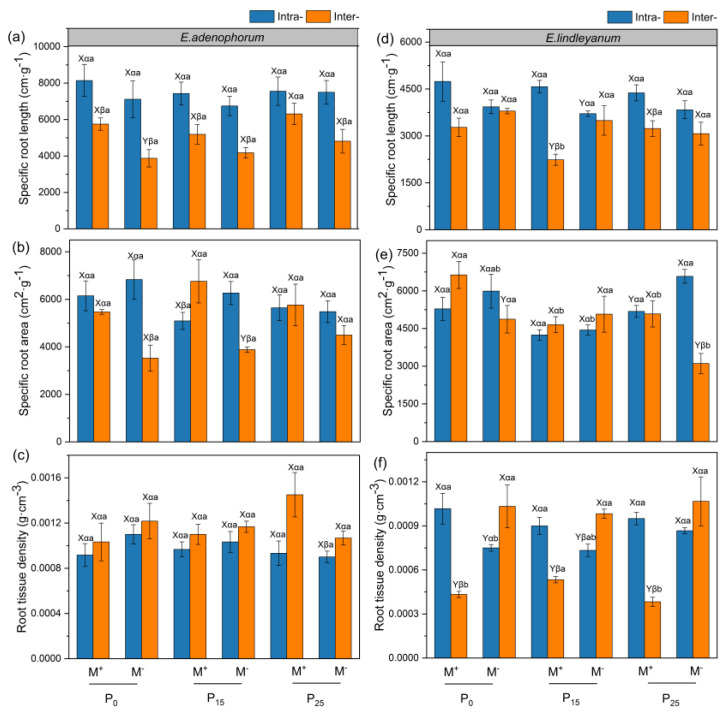
The specific root traits of alien plant *E. adenophorum* and indigenous plant *E. lindleyanum*. The subgraph (**a**–**c**) indicate the specific root length, specific root area, root tissue density of alien plant *E. adenophorum* and subgraph (**d**–**f**) indicate the specific root length, specific root area, root tissue density of indigenous plant *E. lindleyanum*, respectively. Abbreviations: M^+^ = with AMF; M^−^ = without AMF; Intra- = intraspecific, Inter- = interspecific; P_0_ = without P addition of 0 mg·kg^−1^, P_15_ = with P addition of 15 mg·kg^−1^, P_25_ = with P addition of 25 mg·kg^−1^. Different capital letters (X, Y) above the bars indicate significant differences between M^+^ and M^−^ treatments; different Greek letters (α, β) above the bars indicate significant differences between Intra- and Inter- treatments; different lowercase letters (a, b) above the bars indicate significant differences between P_0_, P_15_, and P_25_ treatments (*p* < 0.05).

The AMF treatment had a remarkable impact on the root tissue density of the native *E. lindleyanum*; the competition treatment had a remarkable impact on the specific root traits of alien *E. adenophorum*; the P addition treatment had a remarkable impact on the specific root traits of the native *E. lindleyanum* (Table 1). AMF significantly improved the specific root area of alien *E. adenophorum* in Inter- competition under the P_15_ condition (Figure 5b). For native *E. lindleyanum* under three P conditions, AMF significantly increased the root tissue density in Intra- treatment and significantly decreased the root tissue density in Inter- treatment (Figure 5f). In addition, for alien *E. adenophorum* with M^+^ and M^−^ treatments, the specific root length of Intra- treatment was remarkably higher than Inter- treatment under P_0_ and P_15_ conditions (Figure 5a). For native *E. lindleyanum* with M^+^ treatment, the specific root length and root tissue density of Intra- treatment were higher than Inter- treatment under three P addition conditions (Figure 5d,f). Moreover, for alien *E. adenophorum* with M^+^ and M^−^ treatments, there was no remarkable difference among the three P addition conditions under Intra- and Inter- competition (Figure 5a,c). For native *E. lindleyanum* with M^+^ treatment, the specific root length and specific root area of P_15_ conditions were lower than P_0_ and P_25_ under Inter- competition (Figure 5d,e).

### 2.5. The Root C, N, and P Accumulation of Alien Plant E. adenophorum and Native Plant E. lindleyanum

**Figure 6:**
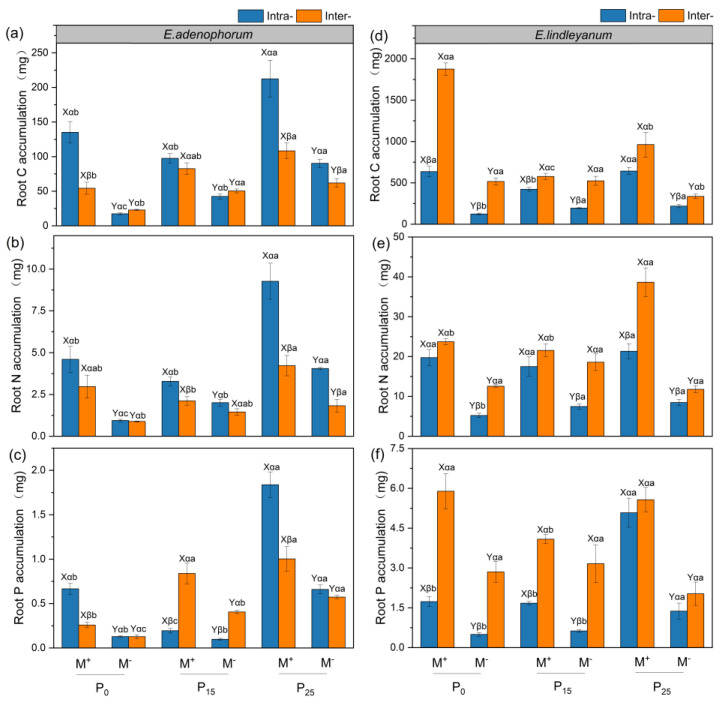
The root C, N, P accumulation of alien plant *E. adenophorum* and indigenous plant *E. lindleyanum*. The subgraph (**a**–**c**) indicate the root C, N, P accumulation of alien plant *E. adenophorum* and subgraph (**d**–**f**) indicate the root C, N, P accumulation of indigenous plant *E. lindleyanum*, respectively. Abbreviations: M^+^ = with AMF; M^−^ = without AMF; Intra- = intraspecific, Inter- = interspecific; P_0_ = without P addition of 0 mg·kg^−1^, P_15_ = with P addition of 15 mg·kg^−1^, P_25_ = with P addition of 25 mg·kg^−1^. Different capital letters (X, Y) above the bars indicate significant differences between M^+^ and M^−^ treatments; different Greek letters (α, β) above the bars indicate significant differences between Intra- and Inter- treatments; different lowercase letters (a–c) above the bars indicate significant differences between P_0_, P_15_, and P_25_ treatments (*p* < 0.05).

As shown in Table 2, AMF remarkably improved the root C, N, and P accumulation of alien *E. adenophorum* in Intra- and Inter- treatments under three P addition conditions and significantly increased the root C, N, and P accumulation of native *E. lindleyanum* in Intra- and Inter- treatments under P_0_ and P_25_ conditions (Figure 6a–f). For alien *E. adenophorum* with M^+^ treatment, the root C, N, and P accumulation of Intra- treatment was higher than Inter- treatment under P_0_ and P_25_ conditions (Figure 6a–c). For native *E. lindleyanum* with M^+^ and M^−^ treatments, the root C, N, and P accumulation of Inter- treatment were higher than Intra- treatment under three P addition conditions (Figure 6d–f). In addition, for alien *E. adenophorum* with M^+^ and M^−^ treatments, the root C, N, and P accumulation of the P_25_ condition were higher than P_0_ and P_15_ conditions under Intra- and Inter- competition (Figure 6a–c). For native *E. lindleyanum* with M^+^ treatment, the root C and P accumulation of P_15_ were lower than P_0_ and P_25_ under Intra- and Inter- competition (Figure 6d–f).

## 3. Discussion

### 3.1. AMF Differently Affected the Root Growth and Nutrition of Alien and Native Plants

In this study, AMF promoted the root biomass, length, surface area, volume, tips, branching points, and root C, N, and P accumulation of alien *E. adenophorum* and native *E. lindleyanum* (Figure 2a,b, Figure 3a–c,e,f, Figure 4a–c,e,f and Figure 6a–f). Many empirical studies have confirmed the function of AMF in promoting host plant growth by assisting the plant root system to efficiently capture nutrients from the soil [39,40,41]. This was consistent with our results, indicating that AMF can promote plants to access more mineral nutrients by altering the root morphological traits of alien and native plants, thus enhancing plant growth and nutrient absorption [3]. Research has demonstrated that differences in the root morphological traits of invaders and natives can represent different nutrient acquisition efficiencies or capabilities [42]. For instance, higher root length, surface area, tips, and branching points mean higher nutrient acquisition ability [43]. Our study suggested that the root morphological traits, such as root length, root surface area, tips, and branching points of native *E. lindleyanum,* were higher than alien *E. adenophorum* (Figure 3 and Figure 4). Therefore, the native *E. lindleyanum* had a greater nutrient acquisition ability than invasive *E. adenophorum*. It was inconsistent with our H1 that AMF promotes the root growth and development of invasive plants more than native plants, resulting in a higher nutrient acquisition ability for invasive plants than native plants. Most previous studies showed that exotic plants had a stronger competitiveness than native plants in growth and nutrition accumulation [44,45]. For one thing, this contradictory result may be attributed to the plant’s biological traits, whereby the native *E. lindleyanum*, as a congener of the invasive *E. adenophorum*, may be more resistant to plant invasion. This finding is demonstrated by a field experiment conducted by Young, Barney [46] in the Central Valley of California, which found that communities containing *Elymus glaucus*, a plant that functions similarly to *Centaurea solstitialis,* were more resistant to invasion than communities lacking functional similarities. For another, this may concern mycorrhizal colonization [45,47]. Studies have shown that the longer the specific root length, the more dependent the plant is on its own root system for nutrients; accordingly, the less dependent the plant is on mycorrhiza [43,48]. Our study held this opinion and demonstrated that the specific root length of invasive *E. adenophorum* was higher than native *E. lindleyanum* (Figure 5), and the root mycorrhizal colonization of native *E. lindleyanum* was higher than invasive *E. adenophorum* (Figure 1). Additionally, some research has shown that plants with a higher root average diameter are more conducive to mycorrhizal colonization [20]. In our research, the root average diameter of native *E. lindleyanum* was greater than invasive *E. adenophorum* (Figure 3d and Figure 4d). Therefore, our results support that native plants congeneric with exotic plants are more resistant to invasion. Further, native plants may develop morphological traits that promote cooperation with mycorrhizae by being more conducive to mycorrhizal colonization and, thus, better resist invasion.

### 3.2. The Competition Differently Affects the Root Growth and Nutrition of Alien and Native Plants

Competition can affect the nutrient absorption of plants by influencing their root morphology [49]. In this study, under M^+^ treatment, the interspecific competition reduced the root mycorrhizal colonization, root growth, and nutrient accumulation of invasive *E. adenophorum* but increased the root mycorrhizal colonization, root growth, and nutrient accumulation of native *E. lindleyanum* relative to the intraspecific competition, indicating that interspecific competition with native *E. lindleyanum* inhibits the root mycorrhizal colonization of exotic *E. adenophorum*, thereby reducing root growth and nutrient acquisition of the invader (Figure 1, Figure 2, Figure 3, Figure 4 and Figure 6). Zhang, Li [32] pointed out that interspecific competition influents the symbiotic relationship between AMF and host plants. Danieli-Silva, Uhlmann [50] showed that the root mycorrhizal colonization of *Cabralea canjerana* decreased, and that of *Lafoensia pacari* increased under interspecific competition. In general, plants with higher root mycorrhizal colonization often receive greater mycorrhizal benefits [51,52]. Therefore, compared to intraspecific competition, interspecific competition allows local plants to gain a greater competitive advantage over invasive plants by reducing the mycorrhizal colonization of invasive plants and improving the mycorrhizal colonization of local plants, which is beneficial for resisting the invasion of alien plants.

### 3.3. Root Traits of Alien and Native Plants Respond Differently to Exogenous P Addition

P is an indispensable nutrient element in plant growth [53], yet, there is very little P in the soil that plants can directly absorb [54]. A previous study has suggested that exogenous P addition decreased the root mycorrhizal colonization [55], which is in line with our findings that the root mycorrhizal colonization of the invasive *E. adenophorum* and native *E. lindleyanum* gradually decreased with exogenous P addition (Figure 1a,b). According to the biological market theory model reported by Wyatt, Kiers [56], when plants can obtain sufficient P directly through their roots, their dependence on AMF is reduced, which indirectly causes a reduction in mycorrhizal colonization. Meanwhile, the plant–fungal symbiosis may be suppressed when sufficient P is provided to the host plant from the outside [57], which is partially consistent with H2 that with exogenous P increase, root mycorrhizal colonization of invasive and native plants is reduced. Additionally, the two plants’ roots responded differently to P addition, showing that root growth, morphological trait values, and nutrient accumulation of invasive *E. adenophorum* increased with P addition, whereas native *E. lindleyanum* reduced with P addition when the two plant species competed (Figure 2, Figure 3, Figure 4 and Figure 6). It was partially inconsistent with our H2 that the two plants’ root growth and nutrition acquisition increase with exogenous P increase. Harpole [58] raised a resource ratio theory for successful invasive plants, suggesting that the nutrient demands of plants differ and that increasing certain nutrient effectiveness may advantage some plants while suppressing the growth of competitors through mutual competition among plants [59]. Our results verify this point, showing that the increase in P addition was beneficial to the root growth of invasive *E. adenophorum* and inhibited the growth of local *E. lindleyanum* root in competition. Compared to exotic plants, native plants have adapted to the local soil nutrient conditions in the long-term adaptation process, and the P addition is equivalent to changing the original soil conditions and may be detrimental to the growth of native plants [60,61]. Notably, the impact of P addition on both plants exists within a range, i.e., the promoting effect of P addition on the alien plant or the inhibiting effect of the native plant does not exceed this threshold, as shown by root growth and development of native *E. lindleyanum* remain higher than that of exotic *E. adenophorum*.

### 3.4. The Interaction of AMF and Exogenous P Addition Differently Affect the Alien and Native Plants

Studies have shown that mutualistic symbiosis with AMF and exogenous P addition can promote plant growth and development, especially in the root system [41,62]. It is consistent with our study, where M × P significantly influenced root length and root C, N, and P accumulation of alien plant *E. adenophorum* and indigenous plant *E. lindleyanum* (Table S1 and Table 2). Furthermore, our research suggested that M × P had no significant impact on the root tips and branching points of native *E. lindleyanum*, while it had a remarkable impact on the root tips and branching points of invasive *E. adenophorum* (Table S1). A high amount of root tips and branches may help invasive *E. adenophorum* to explore nutrients in a larger space [63,64], suggesting that invasive plants may access limited resources by developing lateral roots, i.e., increasing root tips and branches when competing with native *E. lindleyanum*. Previous research showed that the establishment of invasive plants relies on the distinction in nutrient absorption and utilization efficiency between invaders and natives [65], and root traits can reveal plant nutrient uptake and use efficiency [42]. In this experiment, with M^+^ treatment, root growth, morphological trait values, and nutrient accumulation of invasive *E. adenophorum* increased with P addition, whereas native *E. lindleyanum* reduced with P addition when the two plant species competed (Figure 2, Figure 3, Figure 4 and Figure 6), this verifies the H3 of the experiment. The findings indicated that AMF combined with exogenous P addition might exacerbate the invasion of exotic *E. adenophorum*, which was consistent with Zhang, Leng [66], suggesting that long-term fertilization, such as P-fertilizer additions, may lead to greater dominance of exotic species by promoting their growth at the expense of native species. In addition, Lannes, Karrer [67] suggested that increasing P effectiveness can indirectly positively affect invaders. With the development of industry and agriculture, more and more P remains in areas where human activities are frequent, such as farmland and roadsides [68], resulting in the enhancement of soil P level, which may affect the growth of local species and aggravate the invasion of exotic plants. The concern is that, although native plants are higher than invasive plants at different levels of P addition, inoculating AMF and exogenous P addition beyond a certain threshold will reverse the competitive balance between exotic plants and native plants, leading to the successful invasion of exotic plants.

## 4. Materials and Methods

### 4.1. Experimental Material

We selected the alien plant *Eupatorium adenophorum* and the native plant *Eupatorium lindleyanum* as plant materials in this experiment. The invasive *E. adenophorum* is native to Mexico and Costa Rica in Central America and has now widely invaded the southwest of China; the native *E. lindleyanum* is an indigenous plant commonly distributed in the southwest of China. They are both annual herbs of the Eupatorium genus in the Asteraceae family. Seeds of alien *E. adenophorum* and native *E. lindleyanum* were collected in Anshun City, Guizhou Province, China, then brought back to the laboratory and placed in a shaded area to wait for the seeds to fall off naturally. Subsequently, the seeds were stored for experimental use.

The experiment substrate was typical limestone soil collected from the Huaxi district of Guiyang City, Guizhou Province, China. After sieving, we removed the impurities of the root, stone, and leaf from the soil, then sterilized the soil in an autoclave at 0.14 Mpa at 121 °C for 1 h. The soil physicochemical properties were 7.49 pH, 37.14 g/kg organic carbon (SOC), 0.503 g/kg total nitrogen (TN), 410.576 mg/kg available nitrogen (AN), 0.704 g/kg total phosphorus (TP), 5.023 mg/kg available phosphorus (AP), 6.489 g/kg total potassium (TK), and 195.443 mg/kg available potassium (AK). In addition, the experimental fungus inoculum *Glomus etunicatum* was purchased from the Institute of Nutritional Resources, Academy of Agricultural and Forestry Sciences, Beijing. After 4 months of expansion through *Trifolium repens*, the soil was collected and stored in a refrigerator at 4 °C. The presence of fungal spores in the inoculum was observed under a microscope.

### 4.2. Experiment Design

A potting experiment was performed using a plastic pot (22 cm × 20 cm × 28 cm, caliber × bottom diameter × height), and each plastic pot had a round hole of 1 cm in diameter at the bottom to avoid water accumulation. The experiment contained three factors: (1) the AMF treatments included inoculation with (M^+^) and without (M^−^) fungus *Glomus etunicatum* for alien plant *E. adenophorum* and indigenous plant *E. lindleyanum*; (2) the competition treatments involved intraspecific (Intra-) competition, with two alien plant *E. adenophorum* or two indigenous plant *E. lindleyanum* separately planted in a plot, and interspecific (Inter-) competition, with one alien plant *E. adenophorum* plus one indigenous plant *E. lindleyanum* mixed planted in a pot; (3) the P addition treatments included three different P additions, involving no P addition (P_0_), 15 mg/kg P addition (P_15_), and 25 mg/kg P addition (P_25_). The amount of P addition in this experiment was determined according to the Chinese soil nutrient classification standard [69]. Specifically, we dissolved the appropriate amount of sodium dihydrogen phosphate (NaH_2_PO_4_) in 200 mL of distilled water and then sprayed it evenly over the soil surface with a spray bottle. In this experiment, we selected 3–5 seeds of invasive *E. adenophorum* and native *E. lindleyanum* of similar size, disinfected them with 10% hydrogen peroxide for 10 min, and then washed them under distilled water three times. Thereafter, we put approximately 2 kg of sterilized soil into each sterilized plastic pot and then added 50 g of fungus inoculum evenly into the soil. After germination, only two seedlings with good growth were retained in each plastic pot. The remaining plants received P addition once a week for one month. Ultimately, the plant roots were reaped, and the indexes were determined one month after the completion of the P application. The experiment design included two AMF treatments, three P addition treatments, three planting treatments, and six replicates, totaling 108 samples.

### 4.3. Measurements and Calculations

We adopted the method of Giovannetti and Mosse [70] to determine the root mycorrhizal colonization. In addition, we scanned clean plant roots using a digital scanner (STD1600 Epsom, Long Beach, CA, USA; WinRhizo Version410B) to acquire root morphological indicators, such as root length, surface area, volume, average diameter, tips, and branching points. Then, the plant roots were put into sealed bags and then in an oven at 75 °C for drying until the biomass remained unchanged, and the root biomass was recorded through an electronic balance. Subsequently, the specific root length was calculated as root length divided by root biomass; the specific root area was calculated as root surface area divided by root biomass; root tissue density was calculated as root biomass divided by root volume. The measurement of the root C content used the potassium dichromate–sulphuric acid oxidation, and the root N content and P concentration used the diffusion method plus the semi-micro open method and the molybdenum antimony anti-colorimetric [71]. Additionally, the C, N, and P accumulation were the C, N, and P concentrations of plant roots multiplied by the biomass of plant roots.

### 4.4. Statistical Analysis

The experimental data were analyzed by SPSS 27.0 software, and all data were tested for normality and homogeneity of variance before analysis. We used three-way ANOVA to test the effects of AMF (M^+^ and M^−^), competition (Intra- and Inter-), and P addition (P_0_, P_15_, and P_25_) treatments and their interactions on the root mycorrhizal colonization, root biomass, root morphological traits, root C, N, and P accumulation. The significant differences between M^+^ and M^−^, Intra- and Inter-, among P_0_, P_15_, and P_25_ treatments on root mycorrhizal colonization, biomass, morphological traits, and C, N, and P accumulation at 0.05 level were determined with the least significant difference (LSD) test. All figures were produced by Origin 2022.

## 5. Conclusions

We concluded that exogenous P addition reduced the root mycorrhizal colonization of the exotic plant *E. adenophorum*, and the native plant *E. lindleyanum* decreased with increased P addition. AMF significantly promoted the root growth and nutrient accumulation of the two species. When inoculated with AMF, the interspecific competition decreased the root growth and nutrient accumulation of invasive *E. adenophorum* but increased the root growth and nutrient accumulation of native *E. lindleyanum* relative to the intraspecific competition. Meanwhile, the exotic and native plants responded differently to P addition, exhibiting the root growth and nutrient accumulation of invasive *E. adenophorum* increased with P addition, whereas native *E. lindleyanum* decreased with P addition, indicating the exotic and native plants responded differently to P addition. Further, the root biomass, length, surface area, volume, average diameter, tips, branching points, and C, N, and P accumulation of native *E. lindleyanum* were greater than invasive *E. adenophorum* under interspecific competition. Overall, exogenous P addition promoted the invasive plant but decreased the native plant in root growth and nutrient accumulation regulated by AMF, although the native plant outcompeted the invasive plant when the two species competed. The findings may potentially provide evidence that anthropogenically applied P fertilizer increases the possibility of alien plant invasion, providing a perspective to understand the plant invasion mechanisms.

## Figures and Tables

**Table 1:** The three-way ANOVA for the effects of AMF (M^+^ vs. M^−^), competition (Intra- vs. Inter-), and P addition (P_0_ vs. P_15_ vs. P_25_) treatments on the specific root traits of alien plant *E. adenophorum* and indigenous plant *E. lindleyanum*. The *, ** and *** indicate *p* < 0.05, *p* < 0.01 and *p* < 0.001), respectively (the * indicates a significant effect, the ** and *** indicate an extremely significant effect).

Specific Root Traits	Treatments	*df*	*E. adenophorum*		*E. lindleyanum*	
			*F*	*P*	*F*	*P*
Specific root length	M	1	2.569	0.114	0.835	0.364
	C	1	31.377	0.000 ***	13.612	0.000 ***
	P	2	0.938	0.397	11.365	0.000 ***
	M × C	1	0.643	0.426	0.032	0.858
	M × P	2	0.668	0.517	4.637	0.013 *
	C × P	2	0.732	0.485	0.803	0.453
	M × C × P	2	0.212	0.809	5.453	0.007 **
Specific root area	M	1	2.349	0.131	0.369	0.546
	C	1	16.627	0.000 ***	1.056	0.308
	P	2	1.356	0.265	9.109	0.000 ***
	M × C	1	3.166	0.080	33.673	0.000 ***
	M × P	2	0.008	0.992	0.465	0.631
	C × P	2	3.129	0.051	10.872	0.000 ***
	M × C × P	2	0.780	0.463	0.510	0.603
Root tissue density	M	1	0.206	0.651	36.481	0.000 ***
	C	1	11.177	0.001 **	1.052	0.309
	P	2	0.066	0.936	6.205	0.004 **
	M × C	1	1.242	0.270	45.208	0.000 ***
	M × P	2	3.232	0.046	3.997	0.023 *
	C × P	2	0.935	0.398	4.977	0.010 *
	M × C × P	2	0.598	0.553	4.328	0.018 *

**Table 2:** The three-way ANOVA for the effects of AMF (M^+^ vs. M^−^), competition (Intra- vs. Inter-), and P addition (P_0_ vs. P_15_ vs. P_25_) treatments on the C, N, and P accumulation of alien plant *E. adenophorum* and indigenous plant *E. lindleyanum*. The *, ** and *** indicate *p* < 0.05, *p* < 0.01 and *p* < 0.001), respectively (the * indicates a significant effect, the ** and *** indicate an extremely significant effect).

Root Nutrient Accumulation	Treatments	*df*	*E. adenophorum*		*E. lindleyanum*	
			*F*	*P*	*F*	*P*
C accumulation	M	1	119.019	0.000 ***	233.892	0.000 ***
	C	1	33.756	0.000 ***	148.242	0.000 ***
	P	2	36.721	0.000 ***	38.854	0.000 ***
	M × C	1	24.193	0.000 ***	16.552	0.000 ***
	M × P	2	3.962	0.024 *	45.709	0.000 ***
	C × P	2	8.555	0.001 **	32.710	0.000 ***
	M × C × P	2	2.424	0.097	19.514	0.000 ***
N accumulation	M	1	74.996	0.000 ***	125.144	0.000 ***
	C	1	38.926	0.000 ***	51.799	0.000 ***
	P	2	31.699	0.000 ***	8.525	0.001 **
	M × C	1	8.413	0.005 **	0.022	0.882
	M × P	2	6.313	0.003 **	12.032	0.000 ***
	C × P	2	9.122	0.000 ***	2.171	0.123
	M × C × P	2	0.679	0.511	8.623	0.001 **
P accumulation	M	1	70.742	0.000 ***	80.432	0.000 ***
	C	1	3.485	0.067	70.320	0.000 ***
	P	2	61.702	0.000 ***	6.248	0.003 **
	M × C	1	6.470	0.014 *	1.294	0.260
	M × P	2	11.663	0.000 ***	8.267	0.001 **
	C × P	2	24.941	0.000 ***	10.007	0.000 ***
	M × C × P	2	9.075	0.000 ***	1.461	0.240

## Data Availability

All raw data will be available upon request.

## Supplementary Materials

The following are available online at https://www.mdpi.com/article/10.3390/plants12112195/s1, Table S1, The three-way ANOVA for the effects of AMF (M^+^ vs. M^−^), competition (Intra- vs. Inter-), and P addition (P_0_ vs. P_15_ vs. P_25_) treatments on the root biomass and root traits of alien plant *E. adenophorum* and indigenous plant *E. lindleyanum*.
