# Supplementary material for: Invasive and Native Plants Differentially Respond to Exogenous Phosphorus Addition in Root Growth and Nutrition Regulated by Arbuscular Mycorrhizal Fungi
Source: Plants (Basel). 2023 Jun 1;12(11):2195. doi: 10.3390/plants12112195 (PMC10255820; doi:10.3390/plants12112195)
Supplement: Supplementary file 1 [file plants-12-02195-s001.zip › plants-2374892-supplementary.pdf]

Table S1: The three-way ANOVA for the effects of AMF (M^+ *vs.* M^-), competition (Intra- *vs.* Inter-), and P addition (P_0 *vs.* P_{15} *vs.* P_{25}) treatments on the root biomass and root traits of alien plant *E. adenophorum* and indigenous plant *E. lindleyanum*. Abbreviations: M = AMF treatment; C = Competition treatment; P = P addition treatment. The *, ** and *** indicate $P < 0.05$, $P < 0.01$ and $P < 0.001$), respectively (the * indicates a significant effect, the ** and *** indicate an extremely significant effect).

Root traits	Treatments	*df*	***E. adenophorum***		***E.lindleyanum***	
			F	***P***	***F***	***P***
Root biomass	M	1	125.416	0.000***	531.300	0.000***
	C	1	78.585	0.000***	139.815	0.000***
	P	2	28.160	0.000***	20.312	0.000***
	M×C	1	60.921	0.000***	12.049	0.001**
	M×P	2	0.938	0.397	12.239	0.000***
	C×P	2	4.873	0.011*	51.702	0.000***
	M×C×P	2	5.000	0.012*	33.915	0.000***
Root length	M	1	125.416	0.000***	531.300	0.000***
	C	1	626.776	0.000***	104.501	0.000***
	P	2	125.680	0.000***	69.217	0.000***
	M×C	2	12.612	0.000***	50.481	0.000***
	M×P	2	6.580	0.003**	27.195	0.000***
	C×P	2	12.612	0.000***	50.481	0.000***
	M×C×P	2	16.558	0.000***	12.586	0.000***
Root surface area	M	1	235.191	0.000***	875.022	0.000***
	C	1	180.723	0.000***	346.012	0.000***
	P	2	37.517	0.000***	115.006	0.000***
	M×C	1	90.153	0.000***	167.600	0.000***
	M×P	2	0.176	0.839	69.818	0.000***
	C×P	2	12.658	0.000***	125.966	0.000***
	M×C×P	2	11.373	0.000***	55.906	0.000***
Root volume	M	1	769.227	0.000***	1189.482	0.000***
	C	1	741.429	0.000***	324.822	0.000***
	P	2	125.579	0.000***	43.850	0.000***
	M×C	1	489.911	0.000***	199.674	0.000***
	M×P	2	2.736	0.073	38.785	0.000***
	C×P	2	41.194	0.000***	161.935	0.000***
	M×C×P	2	28.444	0.000***	145.227	0.000***
Root average diameter	M	1	2.064	0.156	36.503	0.000***
	C	1	33.931	0.000***	1.584	0.213
	P	2	5.296	0.008**	8.697	0.000***
	M×C	1	7.327	0.009**	7.364	0.009**
	M×P	2	0.290	0.749	5.529	0.006**
	C×P	2	19.700	0.000***	4.640	0.013*
	M×C×P	2	2.496	0.091	1.314	0.276
Root tips	M	1	785.955	0.000***	213.574	0.000***

	C	1	312.050	0.000^{***}	212.563	0.000^{***}
	P	2	129.418	0.000^{***}	6.348	0.003^{**}
	M×C	1	130.756	0.000^{***}	36.801	0.000^{***}
	M×P	2	16.163	0.000^{***}	2.275	0.112
	C×P	2	10.462	0.000^{***}	14.975	0.000^{***}
	M×C×P	2	3.259	0.045^{*}	2.250	0.114
Root branching points	M	1	583.298	0.000^{***}	395.456	0.000^{***}
	C	1	315.762	0.000^{***}	121.715	0.000^{***}
	P	2	0.938	0.397	11.365	0.000^{***}
	M×C	1	129.066	0.000^{***}	3.526	0.065
	M×P	2	10.460	0.000^{***}	2.366	0.103
	C×P	2	14.289	0.000^{***}	10.685	0.000^{***}
	M×C×P	2	9.832	0.000^{***}	7.419	0.001^{**}
